# Near-infrared photoluminescence of Portland cement

**DOI:** 10.1038/s41598-022-05113-1

**Published:** 2022-01-24

**Authors:** Wei Meng, Sergei M. Bachilo, Jafarali Parol, Satish Nagarajaiah, R. Bruce Weisman

**Affiliations:** 1 Department of Civil and Environmental Engineering, Rice University, Houston, TX 77005 USA; 2 Department of Chemistry, Rice University, Houston, TX 77005 USA; 3 Energy and Building Research Center, Kuwait Institute for Scientific Research, 13109 Shuwaikh, Kuwait; 4 Department of Materials Science and NanoEngineering, Rice University, Houston, TX 77005 USA

**Keywords:** Civil engineering, Composites

## Abstract

Portland cement emits bright near-infrared photoluminescence that can be excited by light wavelengths ranging from at least 500–1000 nm. The emission has a peak wavelength near 1140 nm and a width of approximately 30 nm. Its source is suggested to be small particles of silicon associated with calcium silicate phases. The luminescence peak wavelength appears independent of the cement hydration state, aggregates, and mechanical strain but increases weakly with increasing temperature. It varies slightly with the type of cement, suggesting a new non-contact method for identifying cement formulations. After a thin opaque coating is applied to a cement or concrete surface, subsequent formation of microcracks exposes the substrate’s near-infrared emission, revealing the fracture locations, pattern, and progression. This damage would escape detection in normal imaging inspections. Near-infrared luminescence imaging may therefore provide a new tool for non-destructive testing of cement-based structures.

## Introduction

Since its introduction in the early nineteenth century, Portland cement has become an essential component of concrete and related construction materials used around the world. Its global production exceeds 4 × 10^9^ metric tons per year. One of the laboratory tools used to study cement chemical compositions and processes is Raman spectroscopy^1–3^. In this method, samples are irradiated with monochromatic laser light and the scattered light is analyzed for wavelength shifts that reveal vibrational modes characteristic of different chemical components. However, when the laser wavelength is within the visible spectral region, Raman spectra are subject to interference from sample fluorescence. This problem led some cement researchers to shift their Raman excitation wavelength into the near-infrared (near-IR) spectral region^4–6^. The resulting spectra showed odd features that were eventually identified not as the presumed vibrational Raman transitions, but instead as unexpected near-IR luminescence from the cement samples^6^. To date, however, key properties of that unexpected emission have apparently not been investigated. We recently encountered strong and similar near-IR luminescence from specimens while adapting the optical strain sensing method called S^4,7–12^ for use with cement-based materials. Here we report an investigation of that near-IR luminescence and deduce that its origin appears to be crystals of silicon associated with the calcium silicate components in cement. The emission spectra are found to vary with cement type, suggesting a quick, nonintrusive method to distinguish different formulations. In addition, we demonstrate that the strong and spectrally distinct nature of the emission can enable a new scheme for visualizing surface microcracks in concrete structures.

## Materials and methods

We used three commercial Portland cements, Type I/II (from TXI), White (from TXI), and G (from Dyckerhoff AG), to cast specimen blocks with dimensions of 25.4 × 25.4 × 50.8 mm. To compare different hydration states, the Type I/II specimens were made with water/cement ratios ranging from 0.25 to 0.40 and were cured for between 1 and 7 days. We strain tested a Type I/II block with 0.40 water/cement ratio by applying uniaxial compressive stress with a manually actuated vise while monitoring the signal from a resistive foil strain gauge mounted to the specimen.

Photoluminescence spectroscopy involves the capture and spectral analysis of light emitted from a sample when it is optically excited. Our excitation source was a small 660 nm diode laser that was focused onto the surface of cured cement blocks or onto cement powder packed into spectrophotometric cuvettes. The resulting emission was collected by a lens that focused the light into the core of an optical fiber connected to the input of a modular near-IR spectrometer containing a 512-channel InGaAs photodiode array. We supplemented these emission measurements with full excitation-emission scans obtained using a custom-built instrument in which the spectrally filtered beam from a supercontinuum laser provided wavelength-tunable excitation between 500 and 830 nm and a modular InGaAs spectrometer captured near-IR emission spectra. All spectral data were recorded by laboratory computers. Except as noted, measurements were made with samples at ambient temperatures near 23 °C.

## Results and discussion

**Figure 1:**
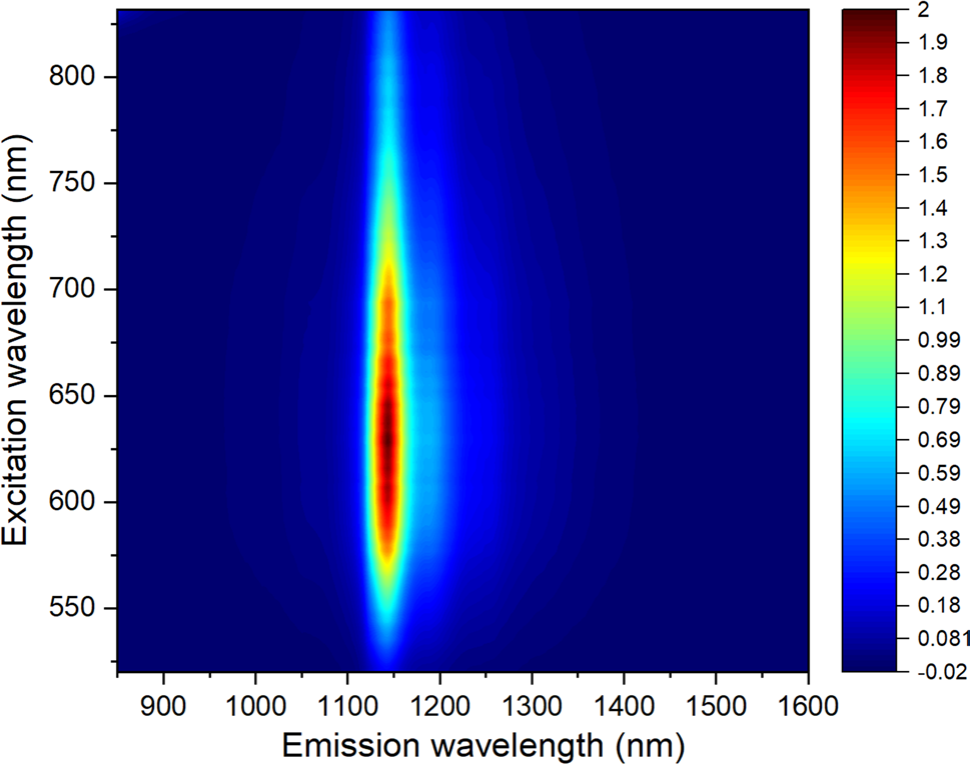
Excitation-emission contour plot for cured Type I/II cement in water. The color scale shows emission intensity in arbitrary units.

**Figure 2:**
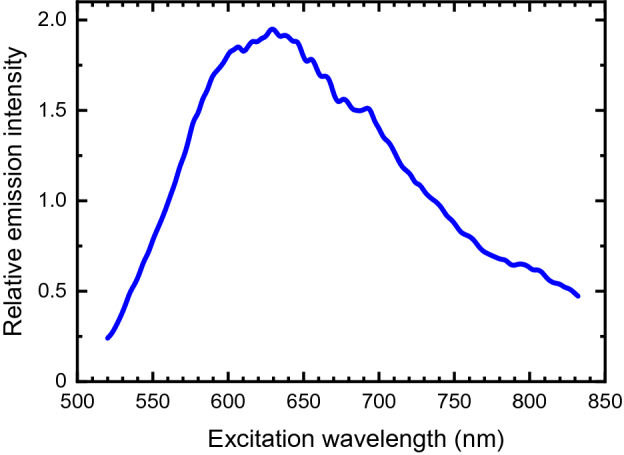
Excitation spectrum for emission at 1140 nm measured for the specimen of Fig. 1.

**Figure 3:**
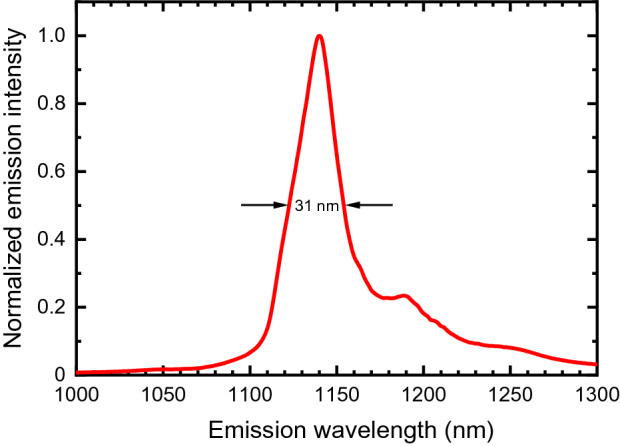
Emission spectrum for the specimen of Fig. 1, excited at 660 nm.

Illumination of a cured block of Type I/II cement at 660 nm gave strong near-IR photoluminescence. Figure 1 displays a contour plot of the excitation-emission map for a sample of Type I/II cement. It shows that the emission profile is independent of excitation wavelength, implying that there is just one luminescent species. Figure 1 also shows that the emission can be induced by an unusually broad range of excitation wavelengths. This is seen more clearly in the excitation spectrum plotted in Fig. 2. Figure 3 displays the much narrower emission spectrum, which has a full width at half-maximum of only 31 nm (30 meV).

**Figure 4:**
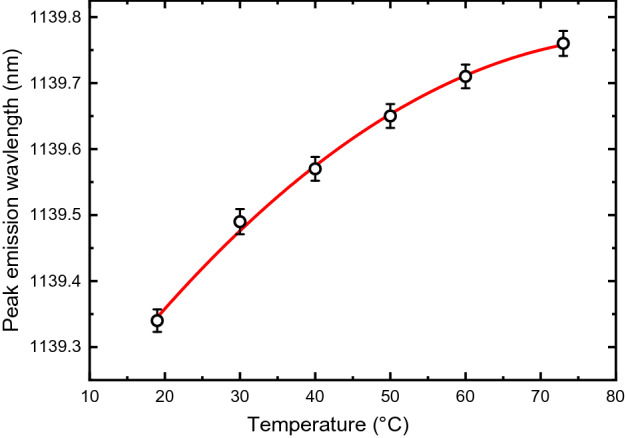
Temperature dependence of the luminescence peak wavelength for a sample of cured Type I/II cement. Points show measured data, error bars show standard errors of the means, and the solid curve is a quadratic fit to the data.

We found that the peak emission wavelengths are the same for cement powder, for cured cement blocks prepared with different water/cement ratios, and for cement blocks cured for different periods (see Supplementary Figs. S1 and S2), so the spectrum is not affected by hydration level. We also observed that the luminescence peak wavelength does not shift as cement specimens are strained: loading a cured specimen to a strain of 500 με gave no measureable peak shift in its emission spectrum. However, we found that temperature does lead to spectral shifts. A cured block was heated on a digital hot plate while spectra were recorded from 121 locations on the specimen surface. As plotted in Fig. 4, the averaged results show that the peak moves systematically to slightly longer wavelength as the sample temperature increases. The emission intensity also decreases by 29% as the sample is warmed from 19 to 73 °C (see Supplementary Fig. S3), suggesting accelerated nonradiative decay.

Our emission spectra may be compared with the cement luminescence artifacts captured in prior near-IR Raman studies of Portland cements and their component minerals. Except for very minor spectral shifts, we find a close match to the Raman artifact in the reported spectrum of monoclinic Ca_3_SiO_5_ (CS3)^5^, a major alite component of cement clinker (the nodular material produced during kilning). The identity of the luminescing species is deduced to be crystalline silicon based on the emission and excitation spectra shown in Figs. 2 and 3. Silicon absorbs throughout the visible spectrum, consistent with our unusually broad excitation spectrum. And like the emission found from cement, silicon’s photoluminescence spectrum has a peak at 1140 nm with a weaker phonon side band near 1200 nm^13–15^. Although silicon’s indirect band gap prevents strong near-IR emission^16^, quantum yields as high as several percent have been reported at room temperature when its nonradiative electron–hole recombination is suppressed by surface texturing and chemical passivation^17^. We suggest that some luminescent crystalline silicon crystals with those surface properties are formed in the calcium silicate phases during high temperature clinker formation. Because of quantum confinement, silicon crystals smaller than ca. 10 nm are known to have band gaps significantly larger than in bulk silicon^14^, with their luminescence shifted to visible wavelengths^15^. This implies that the silicon particles in cement exceed 10 nm in size. We note that the near-IR luminescence observed from Portland cement is at least 100 times more intense than emission from samples of sand or soda-lime glass, two other materials with high silicate contents.

**Figure 5:**
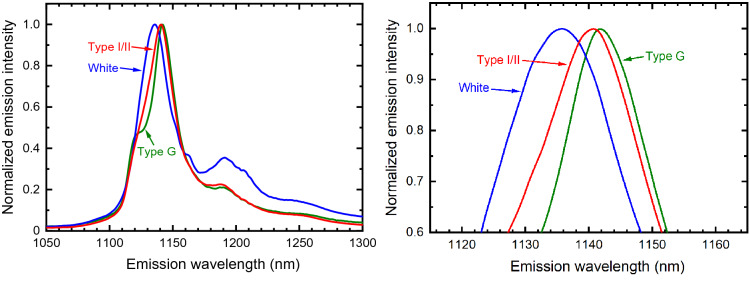
Emission spectra from three cement formulations, with 660 nm excitation. Left panel shows a wide spectral view; right panel shows the same data expanded near the peaks. All specimens were cured and fully hydrated.

Several types of Portland cement are in common use for different application requirements. Among these are the American Society for Testing Materials Types I, II, and White, and the American Petroleum Institute Classes A, B, and G. To spectrally compare some of these, we measured near-IR luminescence from specimens of Type I/II, G, and White cement. The most intense luminescence was observed for White cement (see Supplementary Fig. S4), which has a low content of tetracalcium aluminoferrite. This component therefore seems not to be associated with the near-IR emitters. For each cured specimen, we collected and averaged spectra from 121 points on the surface. Specimens from two different bags of Type I/II cement were measured. The results are plotted in Fig. 5 and the peak wavelengths are listed in Table 1. It can be seen that only a slight difference in peak wavelength was found between the two bags of Type I/II cement, but there were much larger and clear peak shifts among the types. This finding suggests that near-IR emission spectroscopy may find use as a quick, non-contact method to distinguish and identify Portland cement formulations.

**Table 1:** Luminescence spectral peaks from different cement types.

	Type I/II (batch #1)	Type I/II (batch #2)	White cement	Class G
Peak position (nm)	1139	1140	1136	1142

Near-IR cement luminescence may also prove useful for another application. If a specimen is coated with a thin layer of opaque paint and then stressed, small cracks that develop at the surface will propagate outward, breaking the paint layer and exposing the underlying cement-based material. Then when the specimen is illuminated with visible light, characteristic near-IR emission near 1140 nm will be observed only at the site of the fracture. With suitable near-IR imaging, this effect should allow visualization of even very small cracks.

To show proof of concept for this idea, we cast and cured a Portland Type I/II cement block with a 6.4 mm diameter hole through the center as a site for stress concentration during subsequent loading. The specimen was coated with black paint and then compressed along its long axis to induce microcracks near the center hole. To visualize the cracks using intrinsic cement fluorescence, we adapted an apparatus used to map fluorescence emission from strain-sensing carbon nanotube films^16^. This computer-controlled device raster-scanned the surface in a sub-millimeter grid, illuminating each point with 660 nm light and collecting induced near-IR luminescence spectra. By compiling the peak emission signals from the set of scanned points, we obtained a map of luminescence intensity for the 10 × 23 mm^2^ rectangular region of interest near the hole.

**Figure 6:**
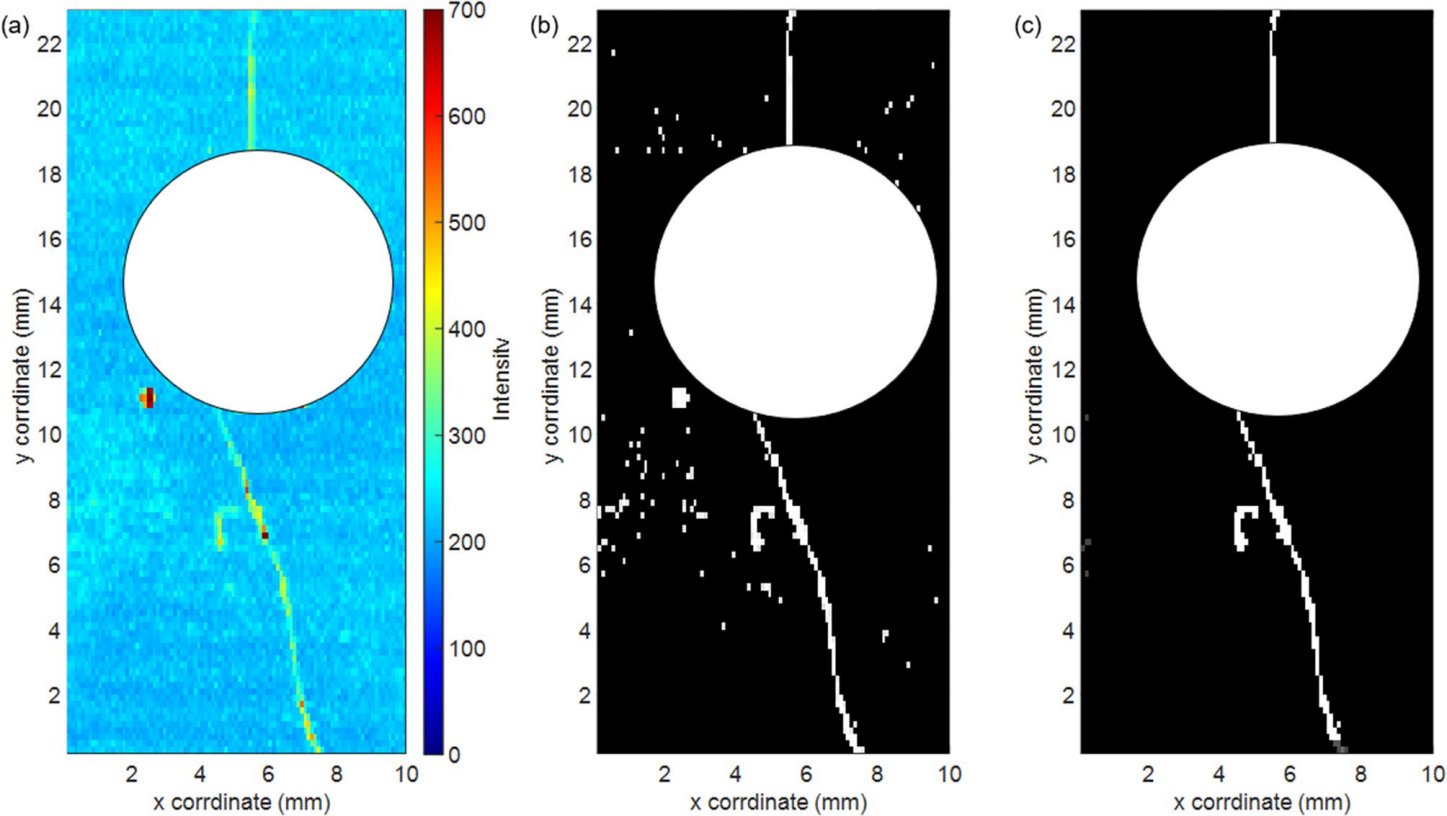
(**a**) Maximum intensity map of the fluorescence emission from the painted cement surface; (**b**) detected cracks after single thresholding; (**c**) detected cracks after hysteresis thresholding, as described in the text.

Figure 6a shows the resulting peak intensity map. To reduce noise and more clearly reveal fractures, we applied a hysteresis thresholding method with two thresholds, *t*_low_ and *t*_high_. Points with an intensity below *t*_low_ were discarded directly and shown as black, while points with an intensity above *t*_high_ were saved and displayed as white. The map after this processing is shown in Fig. 6b. We then used a second step of hysteresis thresholding to further exclude scattered noise points and identify fracture lines^18^. Pixels with intensities between *t*_low_ and *t*_high_ were displayed as white only if any neighboring pixels in the surrounding 3 × 3 region had magnitudes above *t*_high_. If none of the neighbors qualified but at least one fell between *t*_low_ and *t*_high_, then the 5 × 5 pixel surrounding region was searched for pixels with a magnitude above *t*_high_. If that 5 × 5 search was successful, the central pixel was displayed as white; if not, it was classified as noise and displayed as black. Figure 6c shows that this image processing revealed a very clear pattern of microcracks. Microscopic examination of these cracks found widths of only 20 μm, so they would be difficult to detect by normal inspection methods, as illustrated by the conventional photographs of the specimen shown in Supplementary Fig. S5.

A more practical implementation of our crack detection method would involve illuminating an extended area of the specimen surface with visible light and using an InGaAs (near-IR sensitive) camera to image that area through a narrow-band spectral filter that transmits light only near 1140 nm. Compared to crack detection based on normal camera images, this near-IR luminescence imaging approach should greatly increase the optical contrast between fractured and undamaged regions, allowing direct and more sensitive visualization of microcracks that develop on the surface of painted concrete structures.

## Conclusions

We have investigated the unusual near-IR photoluminescence emitted by cement-based materials. The broad excitation spectrum and characteristic emission spectrum of this luminescence point to an origin in silicon crystals, greater than 10 nm in size, associated with the calcium silicate phases of Portland cement. The emission spectra appear independent of cement hydration state or mechanical strain but show a small shift with temperature. Two possible applications of the luminescence are proposed. One is using the small spectral differences found among several types of cement to quickly identify the different formulations. The other application is to image painted concrete surfaces in the near-IR to reveal microcracks that would escape detection in conventional inspections. We suggest that this method may become a useful new tool for the non-destructive testing of critical infrastructure.

## Supplementary Information

Supplementary Information.
