# Supplementary material for: Near-infrared photoluminescence of Portland cement
Source: Sci Rep. 2022 Jan 24;12:1197. doi: 10.1038/s41598-022-05113-1 (PMC8786940; doi:10.1038/s41598-022-05113-1)
Supplement: Supplementary file 1 — Supplementary Information. [file 41598_2022_5113_MOESM1_ESM.pdf]

Supplementary Information

Near-Infrared Photoluminescence of Portland Cement

Wei Meng,[a] Sergei M. Bachilo,[b] Jafarali Parol,[c] Satish Nagarajaiah,[a,d] and R. Bruce Weisman[b,d] *

[a] Department of Civil and Environmental Engineering, Rice University, Houston, Texas, USA 77005

[b] Department of Chemistry, Rice University, Houston, Texas, USA 77005

[c] Energy and Building Research Center, Kuwait Institute for Scientific Research, Shuwaikh, 13109, Kuwait

[d] Department of Materials Science and NanoEngineering, Rice University, Houston, Texas, USA 77005

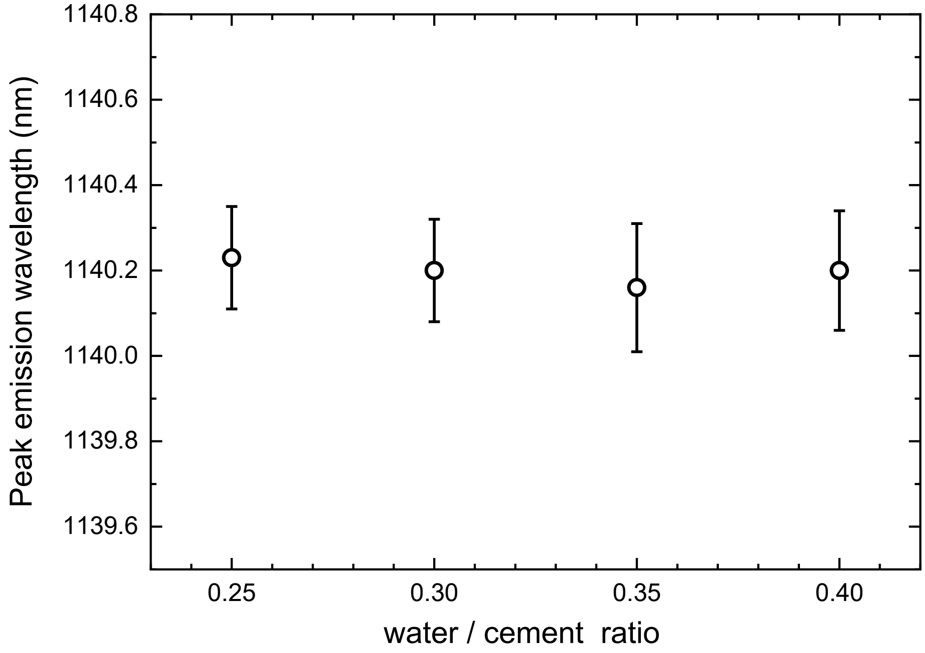

Figure S1. Measured peak emission wavelengths of cured cement samples vs. the water-to-cement ratio used to prepare them.

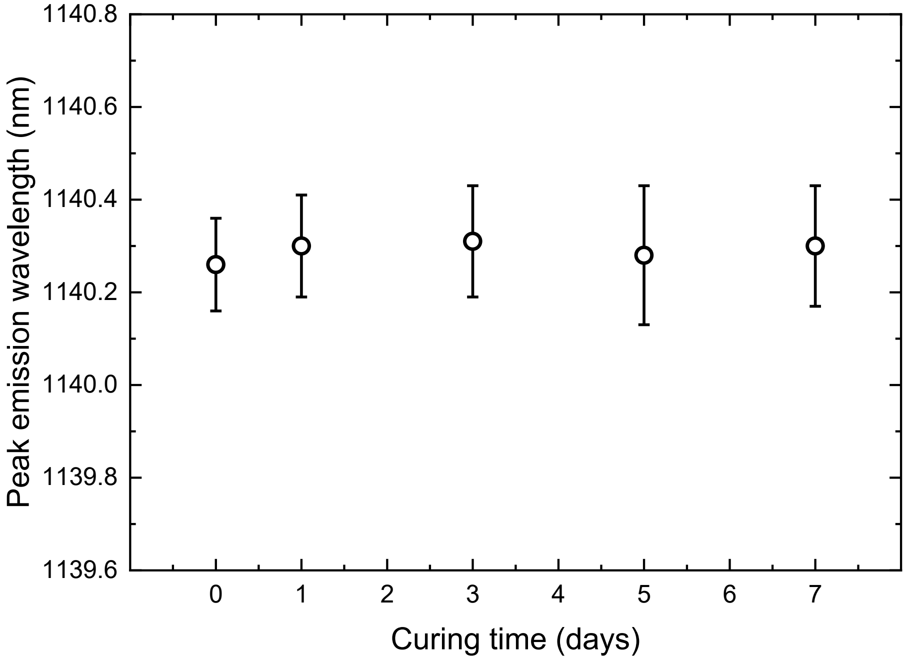

Figure S2. Measured peak emission wavelengths vs. cement curing time. The point at 0 days was measured from dry cement powder.

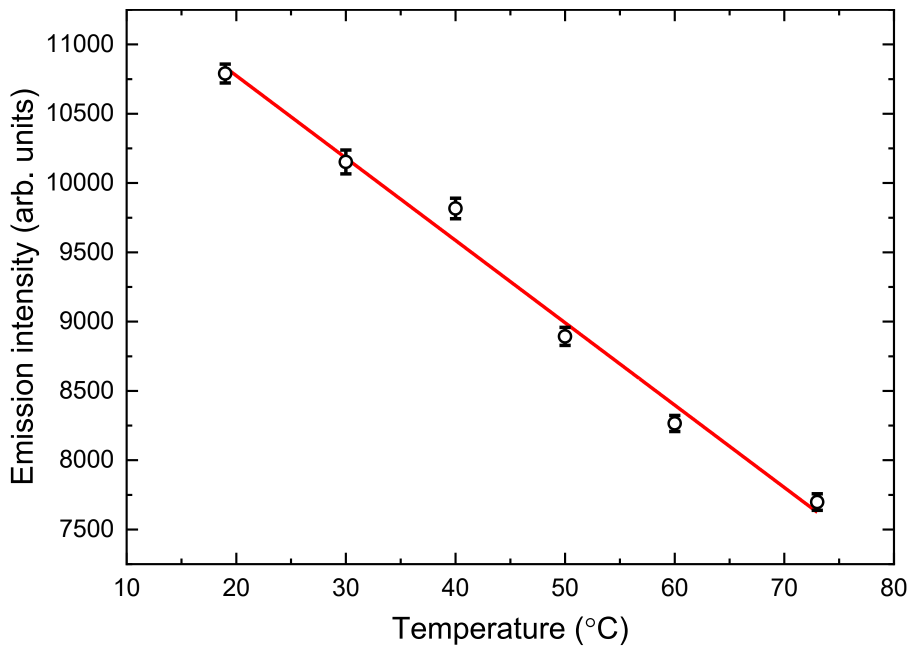

Figure S3. Symbols show measured cement peak emission wavelengths vs. sample temperature. The solid line is a linear best fit to the data.

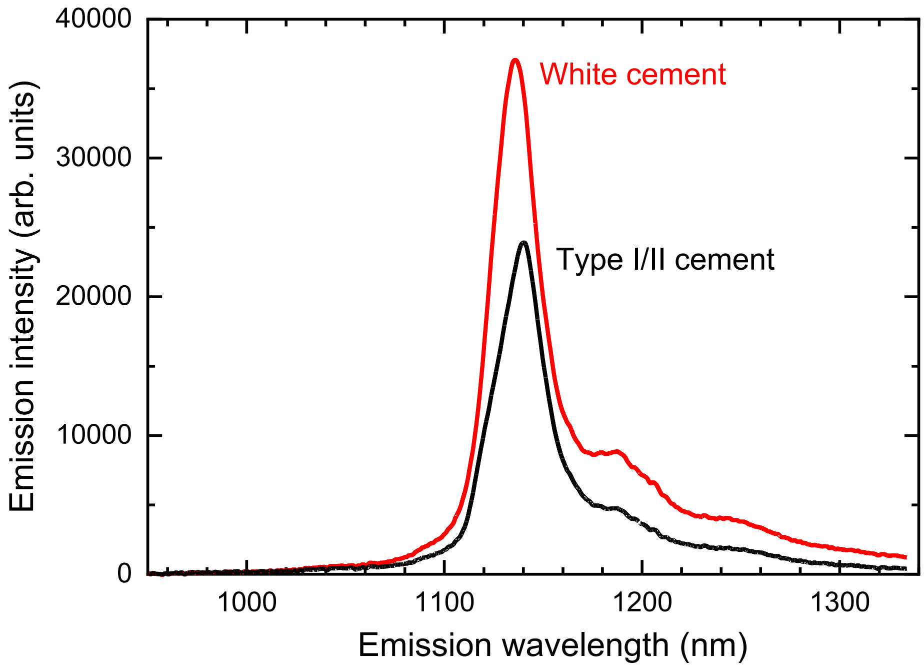

Figure S4. Comparative emission spectra of specimens of White and Grey (Type I/II) cements, measured under the same experimental conditions. The excitation wavelength was 660 nm.

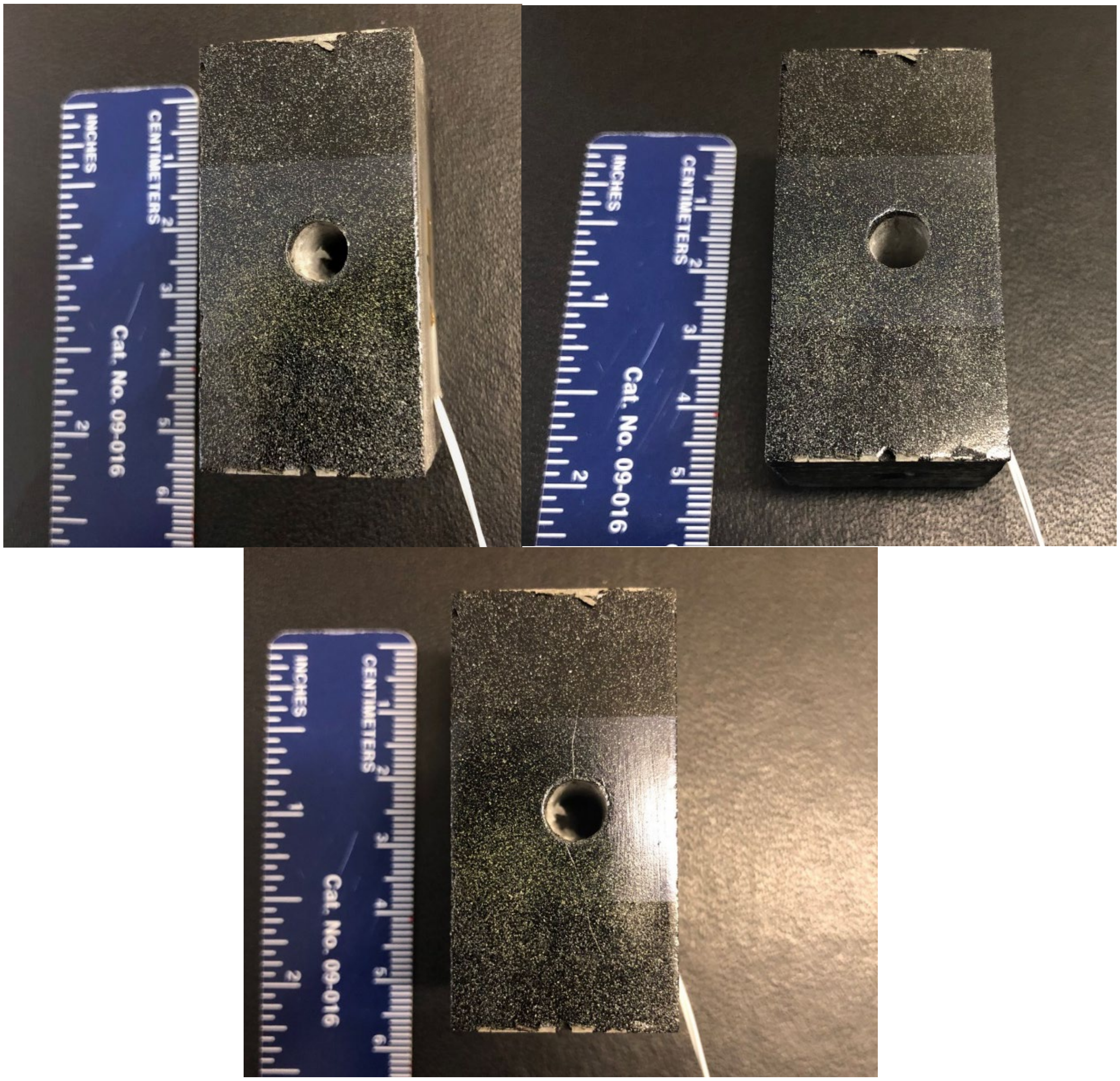

Figure S5. Conventional photographs of a cement specimen that had been coated with black paint and then compressed to develop cracks, as described in the main text and Fig. 6. The three images were captured with different illumination angles. Note that only the bottom image, with illumination at near-normal incidence, reveals the fine cracks estimated to have widths of ~20 μm.
